# The effects of mental practice in neurological rehabilitation; a systematic review and meta-analysis

**DOI:** 10.3389/fnhum.2013.00390

**Published:** 2013-08-02

**Authors:** Susy Braun, Melanie Kleynen, Tessa van Heel, Nena Kruithof, Derick Wade, Anna Beurskens

**Affiliations:** ^1^Research Centre Autonomy and Participation of Patients with a Chronic Illness, Zuyd University of Applied SciencesHeerlen, Netherlands; ^2^School for Public Health and Primary Care (CAPHRI), Maastricht UniversityMaastricht, Netherlands; ^3^Adelante Centre of Expertise in Rehabilitation and AudiologyHoensbroek, Netherlands; ^4^Department for Health and Technique, Physiotherapy, Zuyd University of Applied SciencesHeerlen, Netherlands; ^5^Oxford Centre for EnablementOxford, UK

**Keywords:** neurorehabilitation, mental practice, systematic review, meta-analysis

## Abstract

**Objective:** To investigate the beneficial and adverse effects of a mental practice intervention on activities, cognition, and emotion in patients after stroke, patients with Parkinson's disease or multiple sclerosis.

**Methods:** Electronic databases PubMed/Medline, PEDro, Science Direct, Cochrane Library, PsycINFO, Rehadat, Embase, and Picarta were searched until June 2012. Fourteen randomized controlled trials in stroke and two randomized controlled trials in Parkinson's disease were included, representing 491 patients (421 with stroke). No randomized controlled trials in multiple sclerosis were identified. The methodologic quality of the included trials was assessed with the Amsterdam-Maastricht-Consensus-List (AMCL). Information on study characteristics and outcomes was summarized and evidence for effects described. Data from individual studies in stroke with same outcome measures were pooled.

**Results:** The included 16 randomized controlled trials were heterogeneous and methodologic quality varied. Ten trials reported significant effects in favor of mental practice in patients with stroke (*n* = 9) and Parkinson's disease (*n* = 1). In six studies mental practice had similar effects as therapy as usual (*n* = 5 in stroke and *n* = 1 in Parkinson's disease). Of six performed meta-analyses with identical measures in stroke studies only two showed significant effects of mental practice: short-term improvement of arm-hand-ability (ARAT: *SMD* 0.62; 95% *CI:* 0.05 *to* 1.19) and improvement of performance of activities (NRS: *SMD* 0.9*;* 95% *CI:* 0.04 *to* 1.77). Five studies found effects on cognition (e.g., effects on attention, plan actions in unfamiliar surroundings) and four reported observed side-effects, both positive (e.g., might increase motivation and arousal and reduce depression) and negative (e.g., diminished concentration, irritation).

**Conclusions:** Mental practice might have positive effects on performance of activities in patients with neurological diseases, but this review reports less positive results than earlier published ones. Strengths and limitations of past studies are pointed out. Methodologic recommendations for future studies are given.

## Introduction

Neurological pathologies affect many patients profoundly, causing loss of activities, which often leads to intensive rehabilitation periods (Munneke et al., 2010; Keus et al., 2012). Three often researched neurological conditions of the upper motor neuron are stroke, Parkinson's disease, and multiple sclerosis. The complexity and intensity of neurological multidisciplinary rehabilitation leads to high costs, which will increase as the numbers of patients with a neurological disorder rise (Evers et al., 2004; Struijs et al., 2005; Findley, 2007).

While it is reasonably established that the overall process of neurological rehabilitation is effective, there is little evidence to support many specific rehabilitation therapeutic techniques (Keus et al., 2007a; Langhorne et al., 2009). Currently task orientated practice (i.e., practising a meaningful activity within the context of relevance) and intensity are considered the basis for effective therapeutic techniques (Trombly and Wu, 1999; Langhorne et al., 2009).

Mental practice of tasks is a relatively new therapy that is receiving a lot of attention within rehabilitation research (de Vries and Mulder, 2007; Langhorne et al., 2009). Mental practice can be defined as: *“The repetition or rehearsing of imagined motor acts with the intention of improving their physical execution”* (Malouin and Richards, 2010). Practicing a skill mentally is a potential method to increase the amount of practice during rehabilitation in a safe way with relatively low costs. After initial learning, the mental practice technique can be practiced by the patient independent from the therapist, location, and time of the day.

Over the last decade, many articles investigating the effects of mental practice have been published, including five systematic reviews (Braun et al., 2006; Zimmermann-Schlatter et al., 2008; Nilsen et al., 2010; Barclay-Goddard et al., 2011; Cha et al., 2012). Within neurological rehabilitation, the reviews are restricted to evidence of mental practice in stroke populations. Four reviews focused on upper limb abilities (Zimmermann-Schlatter et al., 2008; Nilsen et al., 2010; Barclay-Goddard et al., 2011; Cha et al., 2012). All reviews included a relatively small number of randomized or clinically controlled trials [four (Zimmermann-Schlatter et al., 2008), five (Braun et al., 2006; Cha et al., 2012), or six (Nilsen et al., 2010; Barclay-Goddard et al., 2011) trials]. The total number of participants on which the evidence was based within the separate reviews ranged from 86 (Zimmermann-Schlatter et al., 2008) to 146 (Cha et al., 2012). All systematic reviews conclude that mental practice might be a potential tool to improve motor functions and activities, but that no definite conclusions on the effects of mental practice can be drawn yet, because the evidence base is relatively small. In addition, the reviews recommend that future research should include identification of who will probably benefit most from mental practice, incorporate follow up measuring points (retention) and investigate whether there are differences in effects of the kind of imagery used (e.g., kinesthetic vs. visual imagery and first vs. third person's view).

Despite the number of recent reviews, there is a need for constant updates of evidence because of the increasing numbers of publications and developments made in this specific area of expertise. Barclay-Goddard et al. (2011) described on-going trials in their Cochrane review in 2011 and estimated that with those studies included the population size on which the evidence would be based would triple (well over 400 participants included). Indeed new studies including some with relatively large sample sizes have been published recently (Ietswaart et al., 2011; Welfringer et al., 2011; Braun et al., 2012; Schuster et al., 2012) and have not yet been included in a review.

Studies assessing the potential of mental practice up until now focused mainly on physical effects. Nilsen et al. (2010) concluded in their review that the variety of effects should be reported more extensively and investigated in future research and Barclay-Goddard et al. emphasize that side-effects, compliance, and integrity should be monitored more closely and reported in future studies (Barclay-Goddard et al., 2011). Mental practice has been shown to regulate arousal, increase control of emotions and improve self-awareness and self-confidence in athletes (Murphy and Jowdy, 1992; Martin et al., 1999) and increase quality of life in patients with breast-cancer (Freeman et al., 2008). At the same time mental imagery may lead to negative side-effects in some patients with complex regional pain syndrome: pain and swelling increased after mental practice use (Moseley et al., 2008).

Although the evidence is yet inconclusive mental practice is recommended to improve arm-hand-abilities in stroke guidelines (Royal College of Physicians of London, 2008; Australian Stroke Foundation, 2010).

Besides in stroke, mental practice has been used in patients with Parkinson's disease and multiple sclerosis. Although it is not possible to compare these target populations in terms of pathology, symptoms, and recovery pattern, the clinical approach for all three patient groups share considerable similarities (e.g., the mental practice instructions given in clinical practice). Within rehabilitation all groups need intensive, task relevant practice. The underlying hypothesis for the value of mental practice is the same: (1) activation of brain regions related to motor function (Johnson, 2000; Cunnington et al., 2001) and (2) increase of intensity of practice without the need to take issues related to safety and physical fatigue into account (Keus et al., 2007b; van Peppen et al., 2007).

The effects of mental practice in Parkinson's disease and multiple sclerosis have not been taken into account in earlier reviews.

The main objective of this study was *to undertake a systematic review and a meta-analysis of randomized controlled trials investigating the beneficial and adverse effects of a mental practice intervention on activities, cognition, and emotion in patients after stroke, patients with Parkinson's disease or patients with multiple sclerosis*.

Strengths and limitations of past studies will be pointed out in order to give recommendations in the discussion section on the content and organization of future trials (Craig et al., 2008).

## Methods

**Figure 1:**
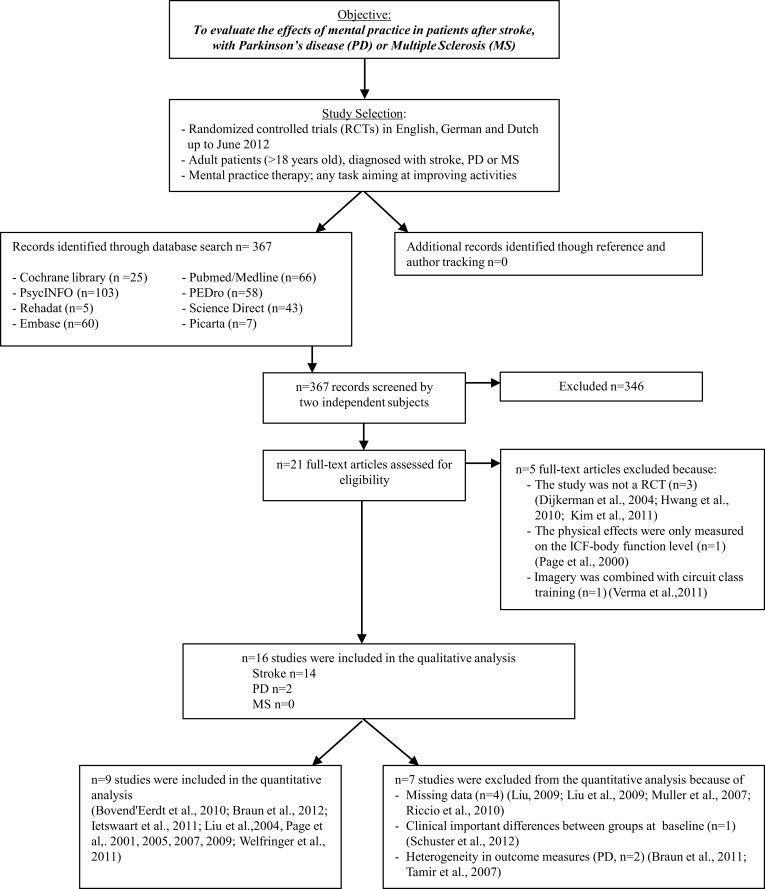
**Overview of literature search**. Abbreviations: MP, mental practice; PD, Parkinson's disease; MS, multiple sclerosis; RCTs, randomized controlled trials.

An overview of the search strategy, selection criteria, quality assessment, and meta-analysis is given in Figure 1.

### Data sources

Computer-aided search was performed by four researchers (Susy Braun, Melanie Kleynen, Tessa van Heel, Nena Kruithof) using PubMed/Medline, PEDro, Science Direct, Cochrane Library, PsycINFO, Rehadat, Embase, and Picarta. The authors hand-searched reference lists of obtained articles (reference and author tracking). Key words used were: *stroke, Parkinson's disease, multiple sclerosis, mental practice, movement and motor imagery, motor learning, rehabilitation, physical therapy, occupational therapy, activities of daily living*. These search terms were used in Dutch and German articles as well and were translated if necessary. The detailed search strategy is available from the authors.

### Study selection

#### Type of study

The studies selected in the review were all available randomized controlled trials in English, German, and Dutch up to June 2012 that reported the effects of mental practice on the improvement of activities during the rehabilitation of adult participants after stroke, with Parkinson's disease or multiple sclerosis. In cross-over trials, only the first phase of the study was taken into account. A study with mixed population was only selected if the majority (over 50%) of participants had been diagnosed with stroke, Parkinson's disease, or multiple sclerosis.

#### Type of intervention

The mental practice intervention could be added to therapy (e.g., using a taped instruction), embedded in therapy (e.g., problem-solving strategies in which overt movements are combined with mental practice during physical or occupational therapy) or given as an independent intervention. Studies in which special equipment was required (such as electro-myographic stimulation and feedback or forms of virtual reality with computer simulation) were excluded. The content of the control intervention should allow the assessment of possible effects of a mental practice intervention.

#### Type of outcome

Outcome measures can be divided into categories according to the international classification of the World Health Organization (ICF; WHO, 2013) of “function” (e.g., a function could be “pain” measured with a “numeric rating scale”), “activity” (e.g., an activity could be “standing up from a chair” measured with a “timed up and go”) and “participation” (e.g., participation could be “providing meals” or “performing (paid) work”). For patients it is important that interventions reduce activity limitations to enable participation in society after returning home. Randomized controlled trials were selected if at least one measure was used for assessing physical effects on the activity level.

### Data extraction and quality assessment

Screening on tittle and abstract was performed by two researchers (Susy Braun, Melanie Kleynen) independently. If based on the information in the abstract, it was not clear whether the study should be included the full-text of the article was assessed.

Methodologic quality assessment of the studies was assessed using the Amsterdam-Maastricht Consensus List for Quality Assessment (AMCL; Van Tulder et al., 2003). The AMCL was originally developed by van Tulder et al. for the Cochrane Collaboration Back Group and includes all criteria of other prominent quality scales like the Delphi List (Verhagen et al., 1998). It rates a study's internal validity and statistical reporting using an 11-point scale (12 criteria), with higher scores indicating higher quality. Each criterion was scored either positive (+, 1 point), negative (−, 0 points), or unclear (?, 0 points), leading to a maximum score of 11 points per study (1 point for the items 2–11; ½ point for the items 1a and 1b).

To increase uniformity in the assessment the validity criteria were defined and then discussed by the two researchers (Susy Braun, Melanie Kleynen). Each item of the AMCL was explained in a separate document that provided uniform operationalization of criteria. In the Appendix the definitions and cut-offs of the criteria of the AMCL are described (Table A1). For example “an acceptable percentage of withdrawals” (criterion 7) was defined as: 10% during the intervention period and from the remaining sample 10% during follow-up as suggested by Van Tulder et al. (2003) Compliance (criterion 5) was considered acceptable if participants themselves or therapists and relatives reported that the participants followed the given instructions. A follow-up period (criterion 10) of at least 3 months was considered clinically relevant for this type of intervention. For these last two mentioned criteria (criteria 5 and 10) reviews of other interventions within health care were used as standard, for generally accepted references in literature were not found (Huibers et al., 2003; Van Tulder et al., 2003). If disagreement on the scores persisted, a third researcher (Anna Beurskens) was approached to reach consensus. A study was defined as being of “sufficient quality” if the score was equal to or above six points. As standard references from the literature are missing, the cut-off was defined by the authors after references from other reviews in physical therapy were taken into account (van Tulder et al., 2001; Van Tulder et al., 2003; Huibers et al., 2003).

Authors of the included articles were contacted to clarify the items on which a question mark was scored. Both scores (blinded assessment as well as after contact with authors) are presented.

Information was extracted from each included trial on: (1) study and population (including number of participants and mean age); (2) type of intervention. We for instance wanted to know if an instruction period for therapists and participants was embedded within the mental practice intervention period (e.g., stepwise approach, tools to check compliance) and what the content of the mental imagery session would be (e.g., what activities were rehearsed and how the imagery was instructed e.g., tape, therapist.); (3) type of outcome measure for physical recovery (primary and secondary measurements, assessment time points, and follow-up period); (4) conclusion (is mental practice recommended and what are the (significant) effects on physical recovery). The conclusions were based on the results and conclusions in the articles but summarized by the independent researchers; (5) All included articles were screened on possibly reported effects on cognition or emotion as well as side-effects (quantitative and qualitative measures). If (secondary) measures were used to consciously search and systematically identify effects on cognition or emotion within the study design the results were categorized as “effects.” Side-effects are described as effects that were not intended, but were observed and reported. These side-effects could be therapeutic (positive) or adverse (negative). Both independent reviewers extracted data from the full papers by using a pre-structured standard form.

### Data synthesis of the meta-analysis

A meta-analysis was conducted using Review Manager version 5.1.6. (The Nordic Cochrane Centre TCC, 2011). Post intervention scores and if possible follow-up scores (at least 3 months) were used. Short- and long-term effects of the intervention were distinguished for two reasons: initial effects might extinguish over time and most studies did not perform a follow-up. Data from both measuring moments were analysed separately. Studies were excluded from the analyses if not all necessary data was provided in the article. No data was imputed.

If a study included two control groups, mental practice was compared to the group with the least chance of improvement (e.g., control group). If no significant differences were found between those groups, it was assumed that no differences would be found between the two experimental groups.

Studies with identical physical effect measurement instruments or studies with instruments measuring the same construct were pooled. The Mean Difference (MD) and 95% confidence interval (95% CI) was used if data was based on identical measurement instruments and the Standardized Mean Difference (SMD) for data based on different measurement instrument. Statistical heterogeneity was assessed using the I^2^-statistic. If I^2^ was greater than 50% outcomes were pooled using SMD with a random effects model. If there was a big variance in Standard Deviations (SD) across studies, reflecting differences in the real variability of outcomes, we also used the SMD. Sensitivity analysis was done to investigate the influence of studies with ACML scores below 6 (lower quality studies). If for instance data were pooled from studies with both lower and high quality, the analysis was performed first with all studies and then repeated without the lower quality studies (Barclay-Goddard et al., 2011).

## Results

In total 367 articles were identified in Pubmed/Medline (*n* = 66), PEDro (*n* = 58), Science Direct (*n* = 43), Cochrane Library (*n* = 25), PsychINFO (*n* = 103), Rehadat (*n* = 5), Embase (*n* = 60), and Picarta (*n* = 7) and 346 were rejected based on title and abstract due to the following reasons: (1) the study was not a randomized controlled trial; (2) the study population did not meet the inclusion criteria; (3) the use of mental practice considered specific equipment; (4) physical effects were only measured on the ICF-body function level; (5) a combination of the reasons above. Of the 21 remaining studies another five articles were excluded after reading the document full-text: one of the studies investigated the effects of mental practice only on the ICF-body function level (only the Fugl Meyer Assessment was used as outcome measure; Page, 2000), three studies were not a randomized controlled trial (Dijkerman et al., 2004; Hwang et al., 2010; Kim et al., 2011), and the last article which was excluded compared imagery combined with circuit class training with Bobath. As the control study did not involve circuit class training, it was unclear what the surplus of the imagery training would be (Verma et al., 2011). Furthermore, the effectiveness and efficiency of circuit training has been established in earlier research (van de Port et al., 2012).

No new articles were retrieved by using reference- and author tracking, leading to a total of sixteen included studies of which 14 in stroke patients and two in patients with Parkinson's disease. No randomized controlled trials with patients with multiple sclerosis were found. In total, 491 participants were included in this systematic review; 421 participants after stroke and 70 participants with Parkinson's disease. The total number of participants in a single study varied from 10 (Page, 2000) to 121 participants (Ietswaart et al., 2011). Group sizes for the experimental intervention varied from 5 to 39 and for the control intervention from 5 to 32 (Page et al., 2009; Ietswaart et al., 2011).

### Effects physical outcome and methodologic quality

The scores on the AMCL (range 0–11 points) of the included studies varied from 3.5 to 8 points after blinded assessment of the reviewers. After additional information was retrieved through authors contact (directly or through earlier confirmed information by the authors in the Cochrane review (Barclay-Goddard et al., 2011)) to clarify the questions marks the scores ranged from 6 to 9 points (Table 1).

**Table 1:**
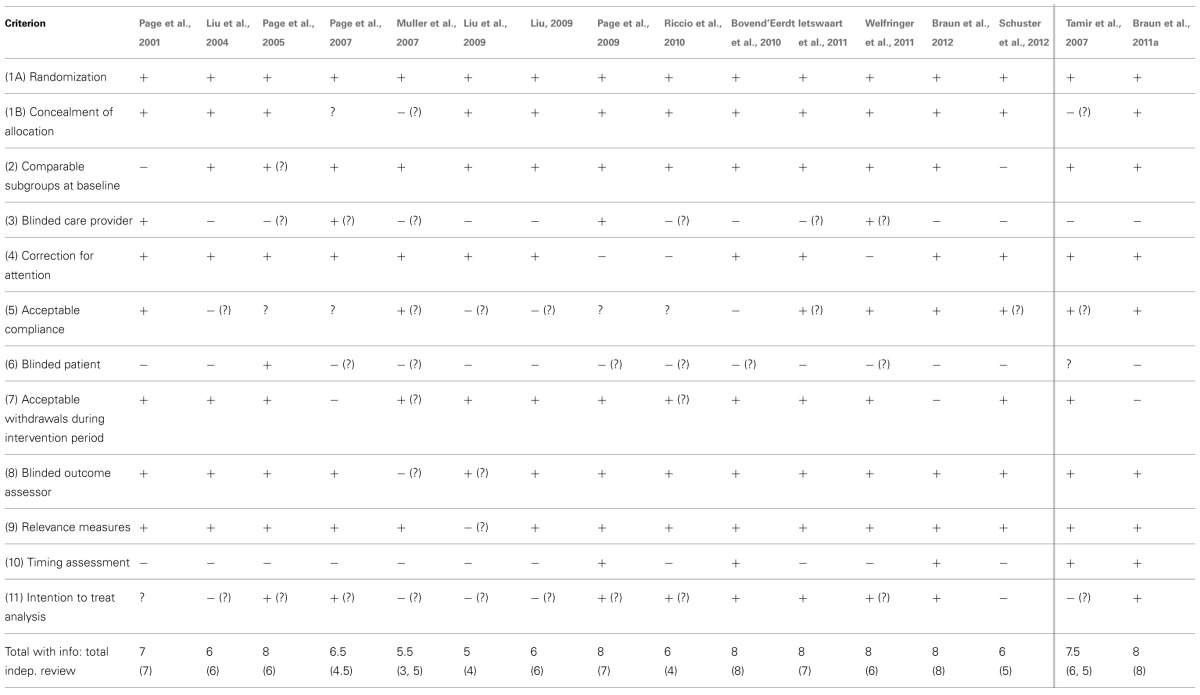
**Quality assessment of internal validity of the RCTs with the AMCL: stroke and Parkinson's disease (PD) population**.

*Each criterion was scored either positive (+, 1 point), negative (–, 0 points), or unclear (?, 0 points). If the score changed after additional information from the authors or earlier confirmed data from other reviews were retrieved, then the initial score from the independent reviewers is shown between brackets*.

*A maximum score of 11 points per study could be achieved (1 point for the items 2–11; ½point for the items 1a and 1b)*.

*The last row of the table shows the total score on the AMCL for all 16 included studies. The top number is the score after additional information from the authors or earlier confirmed data from other publications had been processed. The lower row shows the scores based on information from the original articles only*.

Based on the scores after assessment of the articles by the independent reviewers of the text only, 11 of the 16 studies scored 6 points or more and were considered to have sufficient methodologic quality (Page et al., 2001, 2005, 2009; Liu et al., 2004; Tamir et al., 2007; Liu, 2009; Bovend'Eerdt et al., 2010; Braun et al., 2011a, 2012; Ietswaart et al., 2011; Welfringer et al., 2011). After additional information was processed three more studies came to a total score above six points (Page et al., 2007; Riccio et al., 2010; Schuster et al., 2012). Of these 14 studies with at least sufficient quality, half (*n* = 7) showed overall positive effects of mental practice on arm-hand-function, activities of daily living and mobility of which six in stroke (Page et al., 2001, 2005, 2007, 2009; Liu et al., 2004; Riccio et al., 2010) and one in Parkinson's disease (Tamir et al., 2007). In three high quality studies in stroke positive results were found in favor of the experimental group but not on all outcome measures (Liu, 2009; Welfringer et al., 2011; Schuster et al., 2012) and four high quality studies reported similar effects in the control and experimental group, of which three in stroke (Bovend'Eerdt et al., 2010; Ietswaart et al., 2011; Braun et al., 2012) and one in Parkinson's disease (Braun et al., 2011a). Of the two remaining low quality studies in stroke, one study did not find significant differences between groups (Muller et al., 2007) and one study had mixed results (Liu et al., 2009).

### Effects on physical outcomes in relation to patient characteristics

Study characteristics of the included randomized controlled trials are shown in Table 2.

**Table 2:** **Overview of study characteristics of included RCTs: stroke and Parkinson's disease population**.

**Study**	**Method/population**	**Intervention**	**Measurement instruments, moments and follow-up on physical recovery**	**Conclusion with regard to the effects of MP on physical recovery**	**Effects reported on cognition or emotion reported side-effects**
**STROKE POPULATION**					
Page et al., 2001	Randomized controlled trial	*MP added to therapy*	Primary outcome and primary outcome measure: - ARAT and FM	MP added to therapy may have effects on selectivity and ability of the arm and hand	Interviews and logs were used but showed no side-effects or effects on cognition/emotion
	N total: 13	Both groups: - 6 wk intervention period, 3×/wk 60 min - 30 min upper limb and 30 min lower limb	Timing: - Pretest, posttest 6 wk after the start of the intervention - No follow-up	No test for significance was performed	
	N (EG): 8				
	N (CG): 5				
	Mean age, SD (years): Total: 64.6 ± 14.6	Experimental group: - Daily imagery by tape; 3×/week at home, 2×/week in the clinic - Content tape: 2–3 min relaxation, 7 min MP of ADLs, 2 min refocusing - Three different scripts, 1 for every 2 wk (reaching for and grasping a cup or object, turning a page in a book, proper use of a pencil or pen) - Kinesthetic and visual imagery			
	Time post-stroke (months): EG: range: 2–11 CG: range: 3–11				
		Control group: Tape: 10 min; information about stroke (both at home and in the clinic)			
Liu et al., 2004	Prospective randomized controlled trial	*MP embedded in therapy*	Primary outcome and primary outcome measure: - Patient performance on tasks using a 7-point Likert Scale (trained and untrained tasks)	MP might improve the execution of both trained and untrained tasks	MP group showed significantly greater improvement CTT (subscale) score across time than control group, possibly indicating increased attention and sequential processing
	N total: 46	Both groups: - 3 wk 5×/week for 60 min: PT - 3 wk intervention period, 5×/wk for 60 min with either experimental or control intervention by an OT - Tasks: 15 trained functional tasks including household, cooking and shopping tasks, standardized for all patients of the experimental group - First week easy tasks such as laundry folding, third week: shopping, taking transportation	Secondary outcome and secondary outcome measure: - FM - CTT		
	N (EG): 26				
	N (CG): 20				
	Mean age, SD (years): EG: 71.0 ± 6.0 CG: 72.7 ± 9.4				
	Mean time post-stroke, SD (days): EG: 12.3 ± 5.3 CG: 15.4 ± 12.2	Experimental group: - Daily imagery - First week: analyzing task sequences (motor planning) - Second week: problem identification through MI - Third week: practicing - Kinaesthetic and visual imagery was used	Timing: - Pre-test, post-test 3 wk after the start of the intervention - Follow-up 1 month after post-test		
		Control group: - Daily OT conventional functional retraining program - Same dose as intervention MP			
Page et al., 2005	Randomized controlled trial	*MP added to therapy*	Primary outcome and primary outcome measure: - MAL - ARAT	MP added to therapy as usual may have effects on the use and ability of the arm and hand	–
	N total: 11	Both groups: - 6 wk intervention period, 2×/wk 30 min OT apart from intervention - Task: functional movements of ADLs using affected arm, standardized for all patients of the experimental group			
	N (EG): 6				
	N (CG): 5				
	Mean age, SD (years): Total: 62.3 ± 5.1	Experimental group: - MP by tape after physical practice for 30 min: 5 min relaxation, 20 min MP, 3–5 min refocusing - Internal, cognitive polysensore images were suggested	Timing: - Twice a pre- and once a posttest 6 wk after star of the intervention - No follow-up		
	Mean time post-stroke (months): N total: 23.8 (range: 15–48)	Control group: - Tape: 30 min; progressive relaxation (Jacobson)			
Page et al., 2007	Randomized placebo-controlled trial	*MP added to therapy*	Primary outcome and primary outcome measure: - FM (upper extremity) - ARAT	MP added to therapy as usual may have effects on selectivity and ability of the arm and hand	–
	N total: 32	Both groups: - 6 wk intervention period, 2×/wk 30 min physical therapy apart from MP - Task: functional movements of ADL using affected arm - Three versions: 1 for every 2 wk			
	N (EG): 16				
	N (CG): 16				
	Mean age, SD (years): EG: 58.69 ± 12.86 CG: 60.38 ± 14.17	Experimental group: - MP by tape: 5 min relaxation, 20 min MP, 3–5 min refocusing - Internal, cognitive polysensore images were suggested - 1st person view - Kinaesthetic and visual imagery were used	Timing - Pre-test twice, post-test 1 wk 6 wk after the start of the intervention - No follow-up		
	Mean time post-stroke, SD (months): EG: 38.81 ± 25.86 CG: 45.19 ± 43.56	Control group: - Tape: 30 min; progressive relaxation (Jacobson)			
Muller et al., 2007	Pre-set randomized controlled trial	*MP added to therapy*	Primary outcome and primary outcome measure: - Jebsen Test (slope differences) - Pinch Grip (slope differences)	No differences between the two experimental groups (MOTOR and MENTAL)	-
		All groups: - 4 wk intervention period, 5×/wk 30 min therapy			
	N total: 17	Experimental group1: MENTAL - First session: video-taped finger movement sequence: execute movement until correct order was completed - Thereafter: short refreshment by video followed by only mental rehearsals - Kinaesthetic and visual imagery were used	Timing - 2-week pre-testing - 1-week post-testing 4 wk after the start of the intervention - No follow-up	- Improvement within both experimental groups on all subscales for upper limb use. - Significant differences between both the MOTOR and MENTAL groups on the one hand and the control group on the other in pinch grip and two subscales of the Jebsen Test (“writing” and “simulated feeding”)	
	N (EG1): 6				
	N (EG2): 6				
	N (CG): 5				
	Mean age, SD (years): N total: 62 ± 10	Experimental group2: MOTOR - Perform training task with the affected hand			
	Mean time post-stroke, SD (days): Total: 28.7 ± 21.2	Control group: - Physical therapy			
Liu et al., 2009	Randomized controlled trial	*MP embedded in therapy*	Primary outcome measure: - Performance gains; measuring instrument not mentioned (NRS or Likert Scale)	MP had effect on most of the complex tasks trained: - In 4/5 trained tasks in familiar environment - In 3/5 trained and 2/3 untrained tasks in novel environment	Patients in the MP group seemed to be more able to form cognitive mapping of routes and plan actions in unfamiliar surroundings (effects on cognition)
	N total: 35	Both groups: - 3 wk 5×/week for 60 min: PT - 3 wk intervention period, 5×/wk for 60 min with either experimental or control intervention by an OT - Tasks: 15 trained functional tasks including household, cooking and shopping tasks, standardized for all patients of the experimental group - Each wk, 5 tasks with similar level of difficulty were covered, progressing from the easiest to the most difficult			
	N (EG): 18				
	N (CG): 17				
	Mean age, SD (years) EG: 70.8 ± 9.3 CG: 69.7 ± 7.4		Timing: - Pre-test, post-test 3 wk after the start of the intervention - No follow-up		
	Mean time post-stroke, SD (days): EG: 12.2 ± 5.1 CG: 12.3 ± 7.4	Experimental group: - MP; 5×/wk 60 min - MP involved patients truncating, self-reflecting, feedback, mentally rehearsing combined with performing the activity - Kinaesthetic and visual imagery were used		MP might improve the execution of both trained and untrained tasks	
		Control group: - Control for attention; 5×/wk 60 min (same dose as MP) - Use of a demonstration-then-practice method			
Liu, 2009	Single-blind randomized controlled trial	*MP added to therapy*	Primary outcome measure: - 7-point Likert scale	MP had effect on most of the complex tasks trained: - In 3/5 trained tasks in primary outcome measures in familiar environment - in 5/5 trained tasks in novel environment	No differences between groups were measured with the Cognistat
	N total: 34	Both groups: - 3 wk daily PT for 60 min - 3 wk intervention period, 5×/wk for 60 min with either experimental or control intervention by an OT - Tasks: 15 trained functional tasks including household, cooking and shopping tasks, standardized for all patients of the experimental group - Each wk, 5 tasks with similar level of difficulty were covered, progressing from the easiest to the most difficult	Secondary outcome and secondary outcome measure: - FM - Cognistat		
	N (EG): 17				
	N (CG): 17				
	Mean age, SD (years): EG: 70.4 ± 9.8 CG: 68.1 ± 10.5		Timing: - Pre-test, post-test 3 wk after the start of the intervention - No follow-up		Patients in the MP group seemed to be more able to form cognitive mapping of routes and plan actions in unfamiliar surroundings (effects on cognition)
	Mean time post-stroke, SD (days): EG: 12.3 ± 5.3 CG: 12.3 ± 7.4	Experimental group: - MP: 5×/wk 60 min of which 30 min actually performing the task - MP (30 min) involved self-reflecting and mental imaging - Kinaesthetic and visual imagery were used		MP improved the execution of both trained and untrained tasks. This might indicate that patients in the experimental group were able to generalize learned skills to new situations better than the control group	
		Control group: - Control for attention: 5×/week for 60 min (same dose) - Use of a demonstration-then-practice method			
Page et al., 2009	Randomized controlled trial	*MP added to therapy*	Primary outcome measure: - ARAT - FM (upper extremity)	Larger score changes on the ARAT and FM in the experimental group compared to the control	-
	N total: 10	Both groups: - 10 wk intervention period - Modified constrained-induced therapy (5 h/day, 5 days/wk) - 3 days/wk, 30 min therapy (functional activities)	Timing: - Pre-test twice, post-test 11 wk after the start of the intervention - Follow-up 3 months after the start of the intervention	Differences between groups in favor of the experimental group at post-test but not at follow-up on both the ARAT as FM	
	N (EG): 5				
	N (CG): 5				
	Mean age (years): EG: 58.4 (range: 48–72) CG: 64.4 (range: 56–79)	Experimental group: - MP: 10 wk, directly after therapy (3×/wk 30 min) by tape - 5 different tapes (each for 2 wk) with activities of daily living (practiced in therapy) - Homework: daily cognitive rehearsal - Visual and/or kinaesthetic imagery (patients' preference) was used		No test for significance was performed	
	Mean time post stroke (months): EG: 26.4 (range: 13–45) CG: 30.6 (range: 17–42)	Control group: - Therapy as usual - Tapes were self-administered - Homework on functionally assigned relevant activities was given			
Riccio et al., 2010	Randomized single-blind cross-over study	*MP added to therapy*	Primary outcome measure: - MI (upper limb) - AFT (FAS and time in s)	Group B improved statistically significantly more on all tests at the in-between-assessment	-
	N total: 36	Both groups: - 6 wk intervention period conventional neurorehabilitation 5×/wk, 3 h/day	Timing: - Pre-test, in-between-assessment 3 wk (before cross-over) and post-test 6 wk after the start of the intervention - No follow-up		At post-test, after group A received MP too, no differences between groups existed anymore	
	N (GA): 18				
	N (GB): 18				
	Mean age (years): GA: 60.17 (range 34–75) GB: 60.06 (range 32–75)	Experimental group A: - First 3 weeks only conventional neurorehabilitation - Second 3 weeks MP: 1×/wk 60 min (twice a day 30 min) - MP in a separate quiet room involved relaxation and listening to an audio CD - Activities of the upper limbs, like placing the forearm on the table, were imagined - Kinaesthetic imagery was used		
	Mean time post-stroke (weeks): GA: 7.33 (range 4–12) GB: 7.44 (range 4–12)	Experimental group B: - First 3 weeks MP: 1×/wk 60 min (twice a day 30 min) - MP in a separate quiet room involved relaxation and listening to an audio CD - Activities of the upper limbs, like placing the forearm on the table, were imagined - Kinaesthetic imagery was used - Second three weeks only conventional neurorehabilitation		
Bovend'Eerdt et al., 2010	Single blind randomized controlled trial	MP *embedded* in therapy	Primary outcome measure: - Goal attainment scale	No conclusion: Compliance of therapists and patients was too low	-
	N total: 30	Both groups: - Standard physical- and occupational therapy as usual - 5 weeks intervention period - Homework: from the second half of the intervention period both groups were encouraged to practice at home for at least 5 min/day	Secondary outcome measure: - BI - RMI - TUG - NEADL - ARAT - A custom-developed questionnaire (Imagery Questionnaire) on patient's confidence and perceived effort		
	N (EG): 15				
	N (CG): 15				
	Mean age, SD (years): EG: 62.3 ± 11.75 CG: 50.6 ± 16.48	Experimental group: - MP integrated in therapy - At least 3×/week for first 2 weeks and 2×/weeks for the last 2 weeks (total time imagery ~6.5 h) - A framework for imagery was used - Therapists were trained to teach and monitor MP - Kinaesthetic and visual imagery were used			
	Mixed population: - Stroke: 14 (EG)/14 (CG) - TBI: 0 (EG)/1 (CG) - MS: 1 (EG)/0 (CG)	Control group: - Therapy as usual - Control for attention (same dose)	Timing: - Pre-test, post-test 6 wk after the start of the intervention - Follow-up 12 wk after the start of the intervention		
	Mean time since onset, SD (weeks): EG: 15.9 ± 17.25 CG: 21.8 ± 15.17				
Ietswaart et al., 2011	Randomized controlled trial	*MP added to therapy*	Primary outcome measure: - ARAT	No effects were found on any outcome measure	–
	N total: 121	All groups: - Therapy as usual - 4 weeks of intervention period, 3×/wk 45 min	Secondary outcome measure: - Grip strength, hand-function - BI - Dynamometer, manual dexterity performance - Modified functional limitation profile	An added mental practice intervention has similar effects a controlled therapy as usual	
	N (EG): 39				
	N (placebo CG): 31				
	N (normal care CG): 32				
	Mean age (years): EG: 69.3 Placebo CG: 68.6 Normal care CG: 64.4	Experimental group: - MP: 45 min; 30 min actively imagining (elementary movements, ADL), 10 min active motor imagery (using mirrors and videos), 5 min for a covert form of motor imagery activity (mentally rotating pictures of hands) - Kinaesthetic imagery was used			
	Mean time post-stroke, SD (days): EG: 80.2 ± 55.0 Placebo CG: 90.8 ± 63.4	Attention-placebo control group: - Placebo: 40 min; 25 min active visual and sensory imagery, 10 min cognitive inhibition, 5 min watching optical illusions of motion - Control for attention (same dose)	Timing - Pre-test, post-test 5 wk after baseline - No Follow-up		
	Normal care CG: 80.5 ± 62.7	Normal care control group: - Therapy as usual			
Welfringer et al., 2011	Randomized controlled trial	MP *added* to therapy	Primary outcome measure: - Neglect tests: bells cancellation test, drawing test, and text-reading task	MP had significant effects for the drawing test and the sensation functions only	Negative reported side-effect: - diminished concentration capacity and signs of tiredness at the end training sessions
	N total: 30	Both groups: - Standardized rehabilitation 4×/wk, 45 min - 3 weeks intervention period			
	N (EG): 15				
	N (CG): 15				
	Mean age, SD (years): EG: 56.3 ± 11.2 CG: 57.1 ± 11.3	Experimental group: - MP added to therapy - Two daily half-hour sessions (total 28–30 sessions) - Relaxation, followed by mental practice of positions of the contralesional upper limb: four positions and six sequences (simple and complex movements) - Each exercise up to 10 repetitions - Kinaesthetic imagery was used	Secondary outcome measures: - Representation test (adapted): R-MIQ - Arm-Hand-Function tests: ARAT and sensation functions	Overall test results ambiguous	
				Self-perceived benefits of patients high	Positive reported side-effects: - all patients reported sensations in the left arm during imagery - increased awareness of the left arm
	Mean time post-stroke, SD (months):		General effort to complete a MP session: NRS (1–10)		
	EG: 3.2 ± 1.5	Control group: - No supplementary intervention	Timing: - Pre-test, post-test 3 wk after the start of the intervention - No follow-up		
	CG: 3.4 ± 2.8				
Braun et al., 2012	Multicentre randomized controlled trial	*MP embedded in therapy*	Primary outcome measure: - Numeric Rating Scale (1-10): patients' and therapists' perceived effect on performance of daily activities	No differences between groups short or long-term	Positive side-effect: - Increased feeling of autonomy
	N total: 36	Both groups: - 6 wk intervention period - 6 wk physical therapy; according Dutch multidisciplinary guidelines for stroke rehabilitation - Task: drinking from a cup, walking (standardized) - Optional tasks: self-selected arm and leg activities	Secondary outcome measure: - MI - NHPT - BBS - BI - 10 m-walking test - RMI	An embedded mental practice intervention in therapy as usual in nursing home residents has similar effects as therapy as usual in which there was control for attention	Negative side-effect: - MP costs too much effort to perform (drop out)
	N (EG): 18				
	N (CG): 18				
	Mean age, SD (years): EG: 77.7 ± 7.2 CG: 77.9 ± 7.4	Experimental group: - MP: 6 wk, 5×/wk 30 min - MP was given according to a 4-step framework involving explaining and developing imagery techniques before applying them - Visual and/or kinaesthetic imagery (patients' preference) was used			
	Mean time post-stroke, SD (weeks): EG: 6.1 ± 2.7 CG: 4.8 ± 3.3	Control group: - Therapy as usual - Control for attention: homework (same dose)	Timing: - Pre-test, post-test 6 wk after the start of the intervention - Follow-up 6 months after the start of the intervention		
Schuster et al., 2012	Randomized controlled trial	*MP added to or and embedded in therapy*	Primary outcome measure: - Time difference in seconds to perform the motor task from pre- to post-intervention	No between group differences were found	Logs were used but showed no side-effects or effects outside of the physical domain
	N total: 39	Both groups: - 2 weeks of intervention period - physiotherapy: 6 sessions 25–30 min	Secondary outcome measures: - Help needed to perform the task using CMSA	A mental practice intervention embedded in or added to therapy as usual has similar effects as therapy as usual	
	N (EG1): 13				
	N (EG2): 12				
	N (CG): 14				
	Mean age, SD (years): EG1: 65.8 ± 10.2 EG2: 59.7 ± 13.0 CG: 64.4 ± 6.8	Experimental 1 group (embedded): - MP was embedded in the six sessions - Total intervention time: 45–50 min - PETTLEP-framework was used; physical/emotion, timing, environment, task/learning/perspective - Complete motor task was divided into its 13 stages - Each part was imagined5× before physical performance - Kinaesthetic and visual imagery were used	Achieved stage of motor task - BI - BBS - Computer-based Imaprax questionnaire - KVIQ - Activities-Specific Balance Confidence Scale,		
	Mean time post-stroke, SD (years): EG1: 2.9 ± 1.9 EG2: 4.3 ± 3.6 CG: 3.5 ± 3.9	Experimental 2 group (added): - MP by tape: 3.5 min relaxation, 14.5 min MP, 2 min refocusing. - Total intervention time: 45–50 min - Kinaesthetic and visual imagery were used	intrinsic motivation evaluated in patient's diary		
		Control group: - Physiotherapy - Control for attention: tape; 17 min; 3.5 min relaxation, 11.5 min information about stroke, 2 min refocusing	Timing: - Twice at baseline, before intervention, post-test 2 wk after the start of the intervention - Follow-up after 2 wk		
**PARKINSON'S DISEASE**					
Tamir et al., 2007	Randomized controlled trial	*MP embedded in therapy*	Primary outcome measure: - TUG	MP had significant differences in - TUG - Getting up from a chair or from a supine lying position - Number of steps taken to complete the turn	Cognitive level: + Significant differences in - Stroop test part B indicating an increase in attention and concentration
	N total: 23	Both groups: - 12 wk intervention period, 2×/wk 60 min physical therapy - Protocol of 3 parts (each 15–20 min): (1) callisthenic exercises (2) crucial motor tasks and (3) relaxation exercises - Emphasis on improving the smooth performance of tasks	Secondary outcome measures: - Standing up and laying down - Turning in place 360° - Tandem stance		
	N (EG): 12				
	N (CG): 11				
	Mean age, SD (years): EG: 67.4 ± 9.7 CG: 67.4 ± 9.1	Experimental group: - MP was performed within the second part of the protocol (crucal motor tasks) and during the relaxation period (previously practiced tasks were rehearsed mentally) - Kinaesthetic and visual imagery were used	- Functional reach and shoulder tug - UPDRS	- UPDRS section mental (between group-differences)	(clinical relevance unknown as same amount of improvement in both groups, but an increase in test errors in 4 subjects of the control contrary to 1 in the experimental group)
			Cognitive tasks: - Clock drawing - Stroop test (part A and B)		
	Mean duration of PD, SD (years): EG: 7.4 ± 3.1 CG: 7.8 ± 4.5	Control group: - The exercises were performed physically and relaxation exercises were executed (control for attention, same dose)	Timing: - Pre-test, post-test 12 wk after the start of the intervention - No follow-up		Some indication that imagery might increase motivation and arousal and reduce depression
Braun et al., 2011a	Multicentre randomized controlled trial	MP *embedded* in therapy	Primary outcome measure: - VAS (walking performance), patients' and therapists' perceived effect on performance	No differences between groups at short- or long-term	Negative side-effect: - MP costs too much effort to perform (drop out) - Thinking about motor actions is too confronting (drop out)
	N total: 47	Both groups: - 6 wk intervention period - Physical therapy; 1×/wk 60 min (groups) or 2×/wk 30 min (individuals), according Dutch guidelines for PD - Task: locomotor tasks (walking, standing up from the floor or a chair)		An embedded mental practice intervention in therapy as usual has similar effects as therapy as usual with relaxation	
	N (EG): 25		Secondary outcome measures: - TUG - 10 m walk test		
	N (CG): 22				
	Mean age, SD (years): EG: 70 ± 8 CG: 69 ± 8	Experimental: - MP: 1×/wk 20 min (groups) or 2×/wk 10 min MP (individuals) - MP was given according to a 4-step framework involving explaining and developing imagery techniques before applying them - Visual and/or kinaesthetic imagery (patients' preference) was used	Timing: - Pre-test, post-test 6 wk after the start of the intervention - Follow-up 12 wk after the start of the intervention		
	Mean duration of PD, SD (years): EG: 5.2 ± 5.0 CG: 6.6 ± 7.8				
		Control group: - Control: therapy as usual - Control for attention: progressive relaxation (Jacobson, same dose)			
				

Abbreviations *ADL* *Activities of Daily Living* *AFT* *Arm Functional Test* *ARAT* *Action Research Arm Test* *BBS* *Berg Balance Scale* *BI* *Barthel Index* *CG* *Control Group* *CMSA* *Chedoke-McMaster Stroke Assessment* *CTT* *Color Trails Test* *EG* *Experimental Group* *FAS* *Functional Ability Scale* *FM* *Fugl-Meyer assessment* *h* *hours* *min* *minutes* *KVIQ* *Kinesthetic and Visual Imagery Questionnaire* *MAL* *Motor Activity Log* *MI* *Motricity Index* *MP* *mental practice* *NEADL* *Nottingham Extended Activities of Daily Living* *NHPT* *Nine Hole Peg Test* *NRS* *Numeric Rating Scale* *OT* *Occupational Therapy* *PT* *Physiotherapy* *RMI* *Rivermead Mobility Index* *R-MIQ* *Revised-Movement Imagery Questionnaire* *SD* *Standard Deviation* *TUG* *Timed Up and Go* *UPDRS* *Unified Parkinson's disease Rating Scale* *VAS* *Visual Analogue Scale* *wk* *weeks*.

Age of the participants varied from 40 to 84 years. The time post-stroke differed greatly, ranging from 0 to 7 days after stroke (Liu et al., 2009) to the chronic phase of recovery (>6 months after stroke; Page et al., 2007; Schuster et al., 2012). The average time after the diagnosis of participants with Parkinson's disease was between 5.2 and 7.8 years. Based on these qualitative descriptions mental practice seems to have potential effects in all ages of participants and phases of stroke recovery. In participants with Parkinson's disease, effects of mental practice were more often reported in the two included studies in participants with Hoehn and Yahr stage 1 or 2.

### Effects on physical outcomes in relation to intervention characteristics

Six studies embedded mental practice in therapy (Liu et al., 2004, 2009; Tamir et al., 2007; Bovend'Eerdt et al., 2010; Braun et al., 2011a, 2012), nine studies added mental practice to therapy (Page et al., 2001, 2005, 2007, 2009; Muller et al., 2007; Liu, 2009; Riccio et al., 2010; Ietswaart et al., 2011; Welfringer et al., 2011) and one study investigated both embedded and added mental practice (Schuster et al., 2012).

The intervention in the control group varied from a single intervention like relaxation therapy (Page et al., 2005, 2007), (general) information (Page et al., 2001), embedded therapy as usual (Muller et al., 2007; Tamir et al., 2007; Page et al., 2009; Bovend'Eerdt et al., 2010; Riccio et al., 2010; Braun et al., 2011a, 2012; Ietswaart et al., 2011; Welfringer et al., 2011; Schuster et al., 2012) to therapy according to the demonstration-then-practice method (Liu, 2009; Liu et al., 2004, 2009).

The activities or skills practiced in the intervention group could be restricted to only movements of the arm (e.g., drinking from a cup; Page et al., 2005) or could contain complex tasks involving the entire body (e.g., going to the park; Liu et al., 2009). Frequency of the intervention varied from two to five times a week, while the intervention lasted between 30 and 60 min per session and continued for 2 to 10 weeks. The included studies used different types of imaging (Mulder, 2007): participants were offered kinesthetic motor imagery (Page et al., 2005; Riccio et al., 2010; Ietswaart et al., 2011; Welfringer et al., 2011), or a combination of kinesthetic and visual motor imagery (Page et al., 2001, 2007, 2009; Liu et al., 2004, 2009; Muller et al., 2007; Tamir et al., 2007; Liu, 2009; Bovend'Eerdt et al., 2010; Braun et al., 2011a, 2012; Schuster et al., 2012).

Based on these qualitative descriptions it seems that different kind of interventions may have potential effects on activities (e.g., embedded and added mental practice were both reported effective and ineffective in different studies).

### Effects on cognition or emotion and side-effects

Five studies reported effects on cognition (Liu et al., 2004, 2009; Tamir et al., 2007; Liu, 2009; Welfringer et al., 2011) which were measured with the Stroop test (part B) in participants with Parkinson's disease (Tamir et al., 2007), the Color Trails Test (CTT; Liu et al., 2004) and the Cognistat (Liu, 2009) in participants after stroke (Table 2). Participants with Parkinson's disease seemed to have an increase in attention and concentration after the mental practice intervention period (Tamir et al., 2007). In the studies by Liu et al. (2004, 2009); Liu (2009) the mental practice intervention involved strategy training and participants after stroke seemed to be more able to form cognitive maps of routes and plan actions in unfamiliar surroundings compared to the participants in the control group. Earlier positive findings on the CTT were however not repeated in a later study using the Cognistat (Liu et al., 2004; Liu, 2009).

Positive observed side-effects reported in the stroke trials were increased autonomy (Braun et al., 2012) and increased sensations in and awareness of the left arm (Welfringer et al., 2011). In Parkinson's disease research there was some indication that imagery might increase motivation and arousal and reduce depression (Tamir et al., 2007).

Two studies reported acute adverse side-effects of mental practice (Braun et al., 2011a, 2012) like “too much effort,” “not worthwhile,” and “too confronting.” Some participants after stroke showed diminished concentration and signs of tiredness at the end of mental practice training sessions (Welfringer et al., 2011).

### Data synthesis of the meta-analysis

The meta-analysis was conducted using a selection of the studies in stroke in which the same physical outcome measurement instruments were used (Table 3).

**Table 3:**
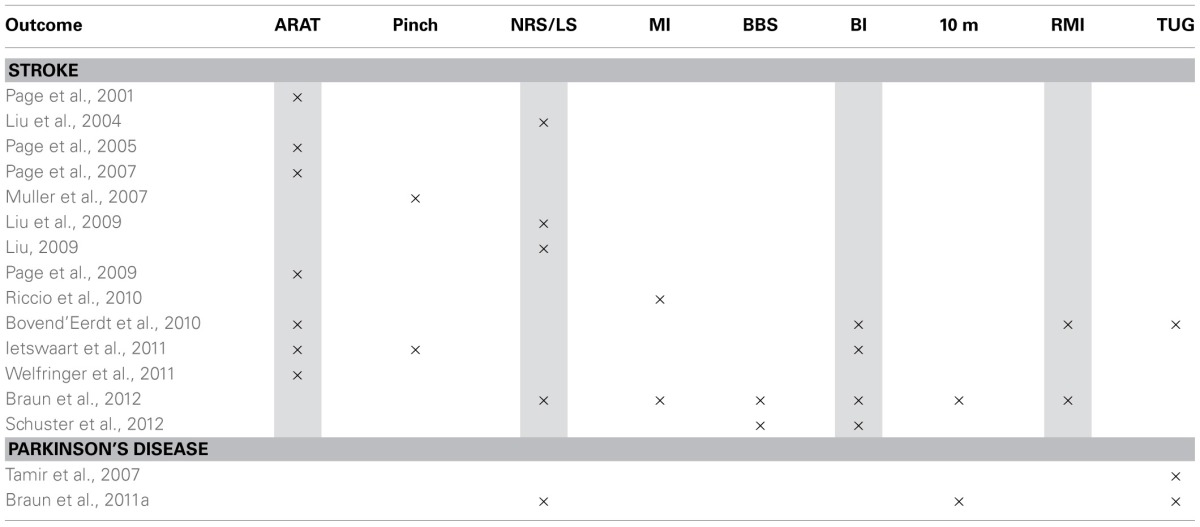
**Overview of used measure instruments that could potentially be used in pooling**.

Abbreviations *ARAT* *Action Research Arm Test* *NRS/LS* *Numeric Rating Scale/Likert Scale* *MI* *Motricity Index* *BBS* *Berg Balance Scale* *BI* *Barthel Index* *10 m* *10 m walking test* *RMI* *Rivermead Mobility Index* *TUG* *Timed Up and Go*.

*Gray Shading: Data could be pooled*.

A meta-analysis in participants with Parkinson's disease was not possible. Both studies use the Timed up and Go as an outcome measure, but Tamir et al. did not report the exact data (only figures provided; Tamir et al., 2007). Several outcome measures which had been used in at least two studies were excluded because of missing data (Motricity Index, Pinch/Hand force; Muller et al., 2007; Riccio et al., 2010). The study of Schuster et al. (2012) was excluded because of clinical important differences between groups at baseline. Six times data could pooled in a meta-analysis. No sensitivity test could be performed as all studies that could be pooled were of at least sufficient quality.

### Results on mobility—rivermead mobility index

Data were available from two studies (Bovend'Eerdt et al., 2010; Braun et al., 2012) that randomized a total of 64 and 58 participants respectively. Pooling did not lead to significant effects assessed with the Rivermead Mobility Index directly after the intervention (*p* = 0.72; *MD*: −0.82; 95%* CI*: −3.04 to 1.41) nor at follow-up (*p* = 0.75; *MD*: −0.40; 95%* CI*: −2.90 to 2.10).

### Results on arm-function—action research arm test (Figure 2)

Data were available from seven studies (Page et al., 2001, 2005, 2007, 2009; Bovend'Eerdt et al., 2010; Ietswaart et al., 2011; Welfringer et al., 2011) that randomized a total of 197 participants. Due to heterogeneity in the SDs of outcomes SMD and random-effect model were used. Pooling led to significant short-term effects on the Action Research Arm Test (*p* = 0.03;*SMD* 0.62; 95% *CI*: 0.05 to 1.19). No data for long-term effects could be pooled.

**Figure 2:**
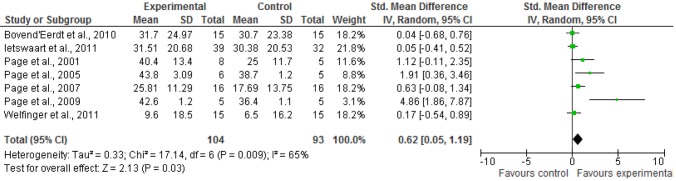
**Results of pooling for ARAT: short-term effects**. Abbreviations: ARAT, Action Research Arm Test; SD, standard deviation; 95% CI, 95% confidence interval.

### Results on functioning in activities of daily living—barthel index

Data of the Barthel Index were available from three studies (Bovend'Eerdt et al., 2010; Ietswaart et al., 2011; Braun et al., 2012) on short-term that randomized a total of 135 participants. Pooling did not show significant effects (*p* = 0.31; *MD*: 0.87; 95% *CI*: −0.80 to 2.53). No significant effects were found at follow-up either (*p* = 0.75; MD:0.46; 95% *CI: −* 2.36 to 3.27). Data for long-term effects were available from two studies (Bovend'Eerdt et al., 2010; Braun et al., 2012) that randomized a total of 57 participants. The study of Liu (Liu et al., 2009) used the modified Barthel Index and was therefore excluded from pooling in both meta-analyses.

### Results from functional activities—numeric rating scale (Figure 3)

Four studies (Liu et al., 2004, 2009; Liu, 2009; Braun et al., 2012) used a numeric rating scale to assess the performance of functional activities. Two studies were excluded from the analyses because they did not provide any point estimates (Liu, 2009; Liu et al., 2009). The data of the studies by Braun et al. and Liu et al. were pooled using SMD because Braun et al. (2012) used a 10-point scale whereas Liu et al. (2004) used a 7-point scale. Liu et al. provide data of the average score of five activities that was used for the analyses. Braun et al. provided scores of the NRS of drinking, walking, and two self-chosen activities. We used the data of the most promising result (biggest different between the experimental and the control group) this was the score of the self-chosen activity for the lower limb. Data of 78 participants could be pooled and a marginal significant overall effect on short-term was found. (*p* = 0.04; *SMD* 0.9; 95% *CI:* 0.04 *to* 1.77). No long-term data could be pooled.

**Figure 3:**
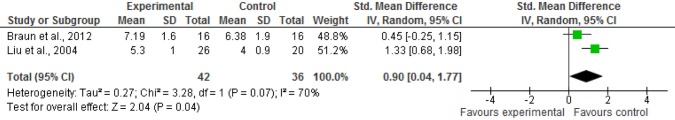
**Results of pooling for NRS: short-term effects**. Abbreviations: NRS, Numeric Rating Scale; SD, standard deviation; 95% CI, 95% confidence interval.

## Discussion

This present review included 16 randomized controlled trials (14 in stroke and two in Parkinson's disease) involving 491 participants (of which 70 in Parkinson's disease) and shows some benefits of a mental practice intervention on arm hand ability (Page et al., 2001, 2005, 2007, 2009; Bovend'Eerdt et al., 2010; Ietswaart et al., 2011; Welfringer et al., 2011) and mobility (Liu et al., 2004; Braun et al., 2012) after stroke. Of the 14 identified studies only 6 showed overall effects in favor of mental practice.

No firm conclusions can be drawn from the existing evidence with regard to the effectiveness of mental practice in participants with Parkinson's disease. No randomized controlled trials within the multiple sclerosis target group were found. Two recently published non-randomized studies investigated the mental practice ability of patients with multiple sclerosis (Heremans et al., 2012a,b). There seems to be a potential use of mental practice in patients with multiple sclerosis. The studies reported in this review remain small (sub groups ranging from 5 to 39 participants), the populations studied vary greatly in most clinical domains, and the outcomes studied also differ a lot. The methodologic quality of the studies ranged from 3.5 to 8 points on the AMCL assessment scale after blinded assessment and also after additional information from the authors was taken into account. This review also finds some evidence for effects on cognition and emotion (e.g., effects on attention, plan actions in unfamiliar surroundings) and reports several observed side-effects (e.g., might increase motivation and arousal and reduce depression, but may also lead to diminished concentration and irritation).

Four recent zero trials (Bovend'Eerdt et al., 2010; Ietswaart et al., 2011; Braun et al., 2012; Schuster et al., 2012) have been added to the body of knowledge on mental practice (*n* = 226), accounting for about half of the total number of participants within all 14 included trials in stroke. Within these zero trials the sample sizes are bigger and more heterogeneous. In addition, more measures on activity level in more general sense were used within these later trials (Barthel Index, BI, and Rivermead Mobility Index, RMI). One could hypothesize that the effects of mental practice are mainly related to aspects as velocity, precision, and coordination of a movement. Improvement in these specific effects of mental practice are perhaps not or hardly detectable with these more generic measures contrary to f.i. the ARAT. In the ARAT and the NRS meta-analyses outcome of both zero and positive trials were pooled, leading to small effect sizes for mental practice in these outcome measures.

Imagery research is still booming and after our search was completed (June 2012) results of new trials were published (f.i. two recent studies on the effects of imagery on gait (Cho et al., 2012; Guttman et al., 2012)). These studies however remain relatively small, but adding new trials to the models could still overturn the results (Langhorne et al., 2009).

### Study limitations

There is a possibility that studies were missed due to inconsistency in terminology used in databases (e.g., mental practice, motor imagery, movement imagery).

The varied clinical populations in this review can be seen as a limitation. This review however does summarize the existing information in neurorehabilitation about a widely used intervention, which will facilitate the exchange of existing knowledge and evidence between professionals working with different target populations. Reviews covering the evidence for specific interventions in multiple target groups, like the recently published one by Newman and Barker (2012) on supported standing, will help professionals get a better understanding of the intervention and potential (side-) effects.

Using assessment scales in general and therefore also the AMCL for rating methodologic quality leads to some practical issues. Blinding of therapists and patients is often not possible in therapeutic interventions like mental practice. If therapists instruct the patients they are not blind to the type of intervention they are providing. The same accounts for the patients when they are asked to actively participate in an intervention. In randomized controlled trials in which therapeutic interventions (e.g., physiotherapy and occupational therapy) are researched the assessment of the randomized controlled trials with any assessment scale will be lower than in for instance pharmaceutical studies. The highest possible score on the AMCL of 11 points will decrease in many therapeutic studies by 2 points, as double blinding is often not possible.

Different assessment tools were used to rate the quality of the included studies (PEDro (Nilsen et al., 2010; Barclay-Goddard et al., 2011), AMCL (Braun et al., 2006), JADAD (Cha et al., 2012)) in earlier reviews. The PEDRO and AMCL are derived from the Delphi list and therefore interrelated (Olivo et al., 2008). The JADAD is a shorter list, most used even though it was not originally developed for therapeutic studies (Olivo et al., 2008). Sometimes the studies within the reviews were categorized into lower and high quality studies (Braun et al., 2006; Zimmermann-Schlatter et al., 2008; Cha et al., 2012) and sometimes the authors of trials were contacted to provided additional information (Barclay-Goddard et al., 2011).

We contacted the authors to clarify the criteria on which a question mark was scored after the blinded reviewing assessment by the independent reviewers was performed. The quality assessment of identical studies may therefore vary within the different reviews as scores on assessment tools normally go up after additional information is retrieved. In one review the quality criteria for assessment of the identified studies were chosen by the reviewers (Zimmermann-Schlatter et al., 2008). These differences make it harder to compare the results and recommendations from the reviews.

Good reporting of trials is important to understanding changes and effects of mental practice and therefore ongoing attention to high quality study reports is required (Barclay-Goddard et al., 2011). Guidelines, like the CONSORT statements are essential to achieve this.

The biggest problem of researching mental practice is the lack of consensus on the definition and concept of the intervention. Heterogeneity within the intervention protocols and outcomes makes it impossible to conduct an overall pooling and thus to come to an overall conclusion.

Results from pooling based on identical outcome measures should be interpreted with caution because of the heterogeneity in study populations. Also, results from meta-analyses depend very much on the data (and models) used. We decided to base the decision on which model to use on the measurement instruments (identical instruments or instruments measuring the same construct) and on the variance in SDs across the included studies. The downside of this flexibility in data/model choice is that it is harder for the reader to follow what has been done in the analysis. The biggest and in our opinion more important advantage is that the outcome is less misleading. Big variation in SDs across studies reflect differences in the real variability of outcomes and the use of MD would in our case suggest potential effects which are probably not there. The study by Ietswaart et al. (2011) with the largest population would for instance have the lowest weight in the meta-analysis and studies with relatively small sample sizes would determine outcome for more than 80%. We tried to correct for this heterogeneity in the analysis and we used change scores instead of effect sizes which might explain to some extend why our results are less optimistic than the meta-analysis by Cha et al. (2012). In the meta-analysis by Cha et al. mental practice combined with exercise therapy had an even bigger effect size (ES 0.51; moderate) then augmented therapy alone (Cha et al., 2012). Two other reviews (Nilsen et al., 2010; Barclay-Goddard et al., 2011) performed statistical analyses to synthesize the evidence of six (Barclay-Goddard et al., 2011) and four (Nilsen et al., 2010) studies. Differences in statistical analysis approaches should be taken into account when interpreting and comparing the results.

Publication bias is a potential weakness in all systematic reviews, as positive or statistically significant findings are more likely to be published than small trials with non-significant or negative findings (Thornton and Lee, 2000). The funnel plot of the ARAT showed indication for publication bias (results not presented) and should therefore be interpreted with care. Barclay-Goddard et al. (2011) identified some risk of bias with regard to concealment of allocation and blinding. Cha et al. (2012) did not report any significant publication bias in their investigation.

### Strengths and limitations of past studies

Determining effects of complex interventions like mental practice is complicated (Braun et al., 2011b). A systematic way of assessing the potential of mental practice could be through the four steps suggested by the Medical Research Council (Craig et al., 2008). Until now, most research has been performed in the first two steps of this model: “determining the working mechanisms” and “piloting.” Fundamental research has shown that mental practice can be performed in patients with neurological conditions and showed that the underlying mechanism is also working in at least parts of the patient populations.

The past 5 years more research has been published on techniques that might assist in monitoring and implementing imagery treatments, like tests (e.g., chronometry, hand-rotation-test; Malouin et al., 2008a; Simmons et al., 2008) and questionnaires (e.g., KVIQ; Malouin et al., 2007, 2008b). Mental practice has been explored in different clinical situations and contexts and a range of different types of intervention, assessed with different measures, have been studied. However, the predictive value of these tests has not been established yet. So we do not know for sure if people who can image according to questionnaires and tests will also benefit from it and whether participants who are at first unable to image, are able to learn and potentially benefit from imagery. In addition, if imagery tests are used as a selection tool patients who are unable to perform these tests are often excluded from research. That is why the study by Welfringer et al. (2011) is of value. Although the results should be interpreted with great caution because participants in the control group did not receive supplementary therapy on top of therapy as usual to control for mental practice, it is until now the only mental practice randomized controlled trial in patients with neglect. Researching feasibility and effects in sub groups that are normally excluded from research will tell us more about whether mental practice can be taught and who might benefit.

There are general methodologic issues in rehabilitations trials (Dobkin, 2007) that also should be considered in mental practice studies. The main problem is that almost all studies are underpowered, increasing the chance of type-2 errors. Especially in mono-centered, small trials the samples are not likely to reflect the real-world sample.

### Recommendations for the design of future trials

Recent negative trials have shown that not all participants with stroke and Parkinson's disease benefit from mental practice. At this point we do not know how to identify the people who might benefit from mental practice. Sample sizes of future trials should be large enough to enable sub group and dose-response analyses. For dose-response analyses adherence, attendance, and compliance should be reported (Barclay-Goddard et al., 2011). Especially adherence and compliance are difficult to assess as mental practice is an intervention that takes place in the mind and remains covert for the therapist. For adherence to mental practice it is essential that participants can engage in movement imagery. However, there is no perfect test to assess this ability. Combining some tests might provide indicators which then might be related to outcome. Therefore, the imagery ability of every participant should be assessed before and/or after the mental practice intervention.

The mental practice intervention should be well described. Both, short- and long-term effects should be measured with predefined measure instruments to enable comparison of results among different studies. Effects should not only be sought at the physical level, but also on emotion and cognition (Nilsen et al., 2010; Barclay-Goddard et al., 2011). Reporting the opinions on and experiences with mental practice of people with neurological diseases, care-givers, and care professionals will provide valuable information on how to optimize and tailor the mental practice intervention to the patients' needs and abilities. Mixed methods are needed to assess these different components.

### Conflict of interest statement

The authors declare that the research was conducted in the absence of any commercial or financial relationships that could be construed as a potential conflict of interest.

## Appendix

**Table A1:** **Amsterdam-Maastricht Consensus List for quality assessment rating criteria**.

**Item**	**Rating criteria**
(1A) Randomization	Item has a positive score if the concealment of treatment allocation is explicitly described to be randomized (e.g., computer generated block randomization)
	Note: Quasi-randomization is scored negative (e.g., randomization of dates of birth or day of the week)
(1B) Concealment of allocation	Item has a positive score if explicitly is described that the allocation of the intervention was blinded (e.g., a independent assessor performs the allocation and has no information/influence on who will be allocated to which group)
(2) Comparable sub groups at baseline	Item has a positive score if the study groups are comparable at baseline with the most important prognostic factors (e.g., comparable mean age and standard deviation in the study groups)
(3) Blinded care provider	Item has a positive score if the care provider is blinded regarding treatment allocation (e.g., the care provider is unaware of the content of the intervention^*^)
(4) Correction for attention; same treatment (dose), co-intervention	Item has a positive score if the different intervention groups have the same treatment dose and if co-interventions are equally divided among the intervention groups. Also, participants in both groups are asked to provide the same information (e.g., fill in logs) and undergo the same tests (battery)
(5) Acceptable compliance	Item has a positive score if participants themselves or therapist and relatives report that the participants followed the given instructions (e.g., through logs, interviews)
(6) Blinded patient	Item has a positive score if patients are blinded regarding treatment allocation and if the method of blinding is appropriate (e.g., the patient is unaware of the treatment content^*^)
(7) Acceptable withdrawals during intervention period	Item has a positive score if the percentage of patients that drop out of the study does not exceed 10% during the intervention period. Another 10% of loss to follow-up of the remaining sample is set as acceptable for the follow-up period
(8) Blinded outcome assessor	Item has a positive score if the outcome assessors are blinded regarding treatment allocation (e.g., independent raters, who are unaware of the treatment group that the participant is in–preferably checked by asking the rater to predict who is in which group)
(9) Relevance measures	Item has a positive score if the measurement instruments allow answering the research question
(10) Timing assessment	Item has a positive score if the outcome assessment takes place approximately at the same time in all intervention groups. Also, a follow-up period of at least 3 months is set to be acceptable
(11) Intention to treat analysis	Item has a positive score if all randomized patients are reported for all measuring points and are analysed according to the group they were originally randomized to

* *Blinding of the care provider and of the patient is not always applicable in physical therapy because of the nature of physical therapy interventions (e.g., manual therapy, exercises). Proper double blinding, therefore, is unlikely to be accomplished for most physical therapy trials (Olivo et al., 2008)*.
